# Lead fixation in deep brain stimulation: comparison of three lead anchoring devices in China

**DOI:** 10.1186/s12893-019-0558-9

**Published:** 2019-07-15

**Authors:** Tao Wang, Yixin Pan, Chencheng Zhang, Shikun Zhan, Bomin Sun, Dianyou Li

**Affiliations:** Department of Functional Neurosurgery, Ruijin Hospital affiliated to Shanghai Jiaotong University School of Medicine, No.197, Ruijin Second Road, Huangpu District, Shanghai, 200025 China

**Keywords:** Deep brain stimulation, Lead anchoring devices, Radiography

## Abstract

**Background:**

The accuracy of deep brain stimulation (DBS) depends on precise electrode positioning, which has been pursued for ideal treatment outcomes. As a critical component of DBS, the fixation performance of lead anchoring devices has been widely studied. Possible reasons for lead shift were analyzed in the current study and we further provided effective solutions to reduce potential manual errors.

**Methods:**

Seventy-nine patients who received DBS implantations at the Ruijin Hospital from April to November 2017 were retrospectively reviewed. Intraoperative lead shifts were measured by C-arm fluoroscopy. Lead adjustment counts were recorded and compared among three lead fixation devices: Stimloc™ (Medtronic, Minneapolis, MN, USA), TouchLoc (SceneRay, Suzhou, China), and the traditional lead anchoring device.

**Results:**

Mean (± SD) distances of lead shifts were 0.29 ± 2.42 mm in Stimloc devices, 0.43 ± 0.55 mm in TouchLoc devices, and 1.52 ± 1.05 mm in traditional devices (*p* < 0.0001). Average numbers of adjustments in this series were 0.3 ± 0.5 in Stimloc devices, 0.3 ± 1.3 in TouchLoc devices, and 1.1 ± 1.0 in traditional devices (*p* = 0.0001). Pairwise comparisons among the three devices (TouchLoc vs. Stimloc: *p* = 0.273; TouchLoc vs. Traditional: *p* = 0.0001; Stimloc vs. traditional: *p* < 0.0001) suggested significant differences, which were mainly attributed to the traditional devices.

**Conclusions:**

Three lead anchoring devices have been compared for their performance in the accuracy of lead fixation, in which the newly designed lead fixation devices have presented its advantages to the traditional one. In addition to the application of the Stimloc and TouchLoc devices, verification by C-arm fluoroscopy should be performed to provide an intuitive view of the depth deviation of electrode position during DBS electrode implantation.

## Background

Deep brain stimulation (DBS) has become a primary neurosurgery for refractory movement disorders, including Parkinson’s disease (PD), essential tremor (ET), and primary dystonia [1]. The accuracy of electrode positioning has always been considered a critical element in this therapy for optimal treatment outcome, and avoiding stimulation-related side effects [2]. Consequently, multiple imaging methods have been applied for measuring the actual position of DBS electrodes and are usually divided into intraoperative and postoperative imaging examinations, including C-arm fluoroscopy, computed tomography (CT), and magnetic resonance imaging (MRI) [3, 4]. With advances in the development of DBS devices, several surgical steps have already been optimized to reduce the potential for lead shifts. One of the steps is the lead anchoring device, which has been used for securing DBS leads onto the cranial burr hole for more than one decade [5, 6].

With the rapid development of DBS in China over the past two decades, three DBS manufacturers (Medtronic [Minneapolis, MN, USA], PINS Medical [Beijing, China], and SceneRay [Suzhou, China]) have offered several DBS products, among which patented lead anchoring devices were undoubtedly included for the optimization of this surgery [7]. Although the appearance and specifications of these lead anchoring devices are not exactly the same, the key application of lead fixation has become vitally important to the accuracy of this therapy, which is usually verified intraoperatively by the imaging methods mentioned above. Unfortunately, intraoperative CT and MRI are not universally available in all Chinese functional neurosurgery centers. Instead, C-arm fluoroscopy has played a practical role in intraoperative confirmation of lead placement because of its accessibility and reliability [4, 8]. So far, few literatures have described differences in lead fixation performance among lead anchoring devices. Such a comparison would facilitate surgical optimization and accuracy improvement in DBS rather than promoting better lead anchoring devices. Aside from providing an analysis of lead fixation performance among these three lead anchoring devices, we also present a surgical technique for lead anchoring in an attempt to reduce artificial errors based on our abundant experiences with DBS surgeries in our center (>2000 dB leads implanted in the past decade).

## Methods

### Participants

This retrospective study reviewed 79 consecutive patients treated between April and November 2017 at the Ruijin Hospital, affiliated with Shanghai Jiaotong University School of Medicine (Shanghai, China). These patients received DBS treatments for intractable movement disorders (PD, ET and dystonia) as well as refractory mental disorders (obsessive-compulsive disorder, anorexia nervosa, drug addiction and major depressive disorder) from neurosurgeons in the interdisciplinary team. Lead positions were all confirmed through routine intraoperative C-arm fluoroscopy by at least two neurosurgeons in our center. Informed written consents were provided by all patients before surgery, and the use of anonymized patient data was approved by the Ethics Committee of Ruijin Hospital, affiliated with the Shanghai Jiaotong University School of Medicine.

### Lead anchoring devices

**Fig. 1:**
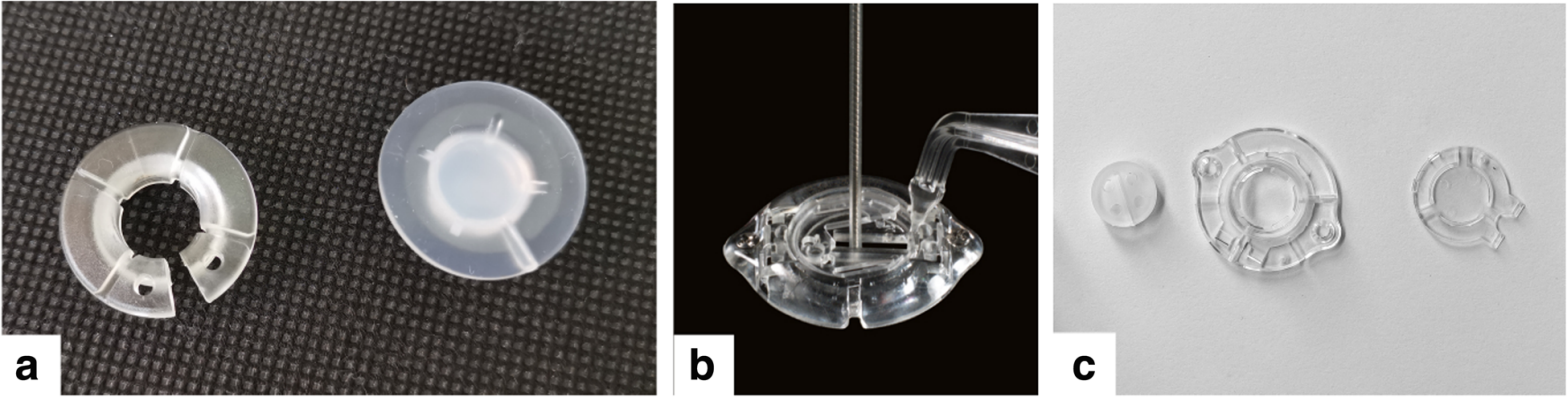
Three lead anchoring devices used in this study. **a**, traditional lead anchoring device: the left part is the base ring which will be installed on the burr hole, and the right part is the cap to seal the hole and press the lead to the groove on the base ring; **b**, Stimloc™ (Medtronic, Minneapolis, MN, USA) lead anchoring device: the lead is fixed in the middle of the base ring by the built-in clamp with the assistant of the handle; **c**, TouchLoc (SceneRay, Suzhou, China) lead anchoring device: the left part is the clamp to lock the lead, which will be installed in the base ring (the middle one) with the cap (the right one) to seal the burr hole

Lead anchoring devices in this study were from three DBS manufactures in China including Medtronic, PINS Medical, and SceneRay. The traditional lead anchoring devices were used in the DBS products manufactured by Medtronic and PINS Medical, while the Stimloc™ and TouchLoc devices were exclusively owned and patented by Medtronic and SceneRay, respectively (Fig. 1). Specifically, the traditional one simply consists of the base ring installed on the burr hole and the cap to press the lead on the groove of the base ring without the lead clamp, which is not patented as the above two.

### Surgical protocol

**Fig. 2:**
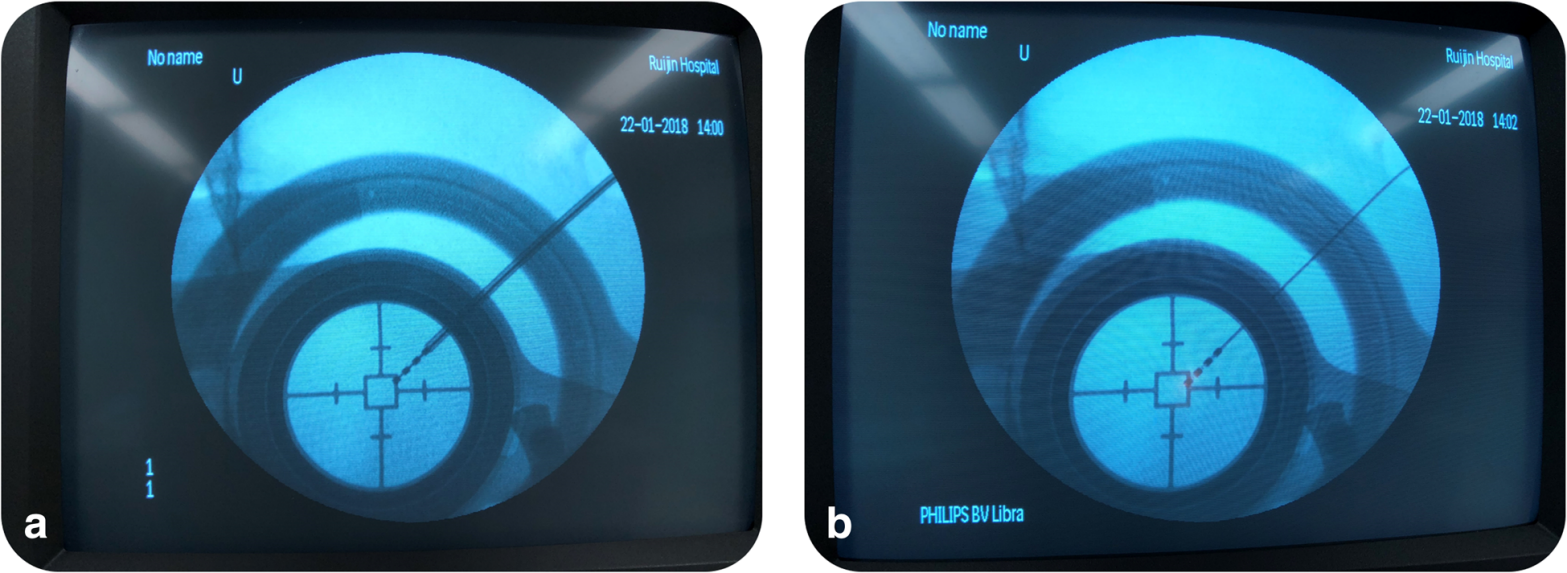
Lead shift imaging using C-arm fluoroscopy. **a**, Lead position after implantation. **b**, Lead position after pressing the lead onto the groove of the dock

The standard protocols of DBS surgery were used and have been described in previous articles by the authors [9, 10]. Briefly, after target planning and general anesthesia, with the patient in supine position, a burr hole was formed at a predetermined location according to target coordinates. It was important that the center of the hole was located at the entry point of the DBS lead as precisely as possible. Following burr hole formation, the lead anchoring device was attached over the burr hole and fixed to the skull to secure the cannula after DBS lead implantation. Subsequently, electrode position was confirmed by C-arm fluoroscopy for the first time, in which the position was measured by the distance from the distal contact of the electrode to the reference bar attached to the DBS frame. Once the guidewire of the DBS lead and the cannula were removed, the lead was carefully pressed by the surgeon on the fillister of the anchoring device. A second C-arm fluoroscopy was then used to compare electrode positions in the two images. The comparison revealed the relative shift in the two images and was measured in the length of the electrode contact, as a result of which, the minimum measurement unit was 0.5 mm (Fig. 2). Adjustment of the depth of the lead continued until the difference between the actual and the original positions were within the range of acceptable error (0.5 mm). The number of lead adjustments in electrode positioning were also recorded in this study. When finishing adjustments, the cap of the anchoring device was covered and locked on the lead firmly. Reconfirmation by using C-arm fluoroscopy was cautiously performed when using the traditional lead anchoring device. The maximum difference was then recorded to be the lead shift in each implantation, while the relevant adjustment and the imaging verification of electrode position were indispensable in the following steps. Once the lead was located at the planned site, the remaining portion was then inserted subcutaneously and coiled around the base ring, followed by wound suture and the implantation of an internal pulse generator.

### Statistical analysis

The main outcome measures in this study were the distances of lead shifts and lead adjustment counts. A positive value of shift distance meant that the actual position was deeper than planned, while a negative value meant the opposite. In addition to descriptive statistics of patients’ demographic characteristics, the Kruskal-Wallis test was performed as a nonparametric alternative for a one-way analysis of variance (ANOVA) of the three lead anchoring devices in view of non-normal distribution of lead shift distances and adjustment counts. Then pairwise comparisons were performed. All statistical analysis was performed using SPSS version 23.0 (IBM Corporation, Armonk, NY, USA), and the descriptive results were presented as mean ± standard deviation (SD); the significance level was set to 0.05.

## Results

### Patient characteristics

This retrospective study reviewed 79 patients (41 men, 38 women; mean age 56.8 ± 16.2 years [range, 17–79 years]) who underwent DBS surgery (Table 1). Among the lead anchoring devices, 28 patients received the Stimloc™, 25 received the TouchLoc, and 26 received the traditional lead anchoring devices. In the traditional device group, 6 patients received devices manufactured by Medtronic and the remaining received products manufactured by PINS Medical. These patients experienced movement disorders including: PD (*n* = 49); Parkinsonian syndrome (*n* = 3); ET (n = 4); and primary/secondary dystonia (*n* = 14). Mental disorders were discovered in 1 patient with obsessive-compulsive disorder, 1 with anxiety disorder, 3 with drug addiction, 2 with tic disorder, and 2 with major depressive disorder. Accordingly, the electrodes were implanted in several functional nuclei, including 8 electrodes in the subthalamic nucleus (STN) of 6 patients, 123 electrodes in the globus pallidus internal (GPi) of 66 patients, 10 electrodes in the ventral capsule/ventral striatum of 5 patients, 4 electrodes in the habenular nucleus of 2 patients, 4 electrodes in the posterior subthalamic area of 2 patients, and 4 electrodes in the ventralis intermedius nucleus of 2 patients. Among these, 5 patients received unilateral DBS surgeries because of unilateral symptoms (*n* = 4) or reoperation for lead location modification (*n* = 1). All surgeries were performed under general anesthesia, except in 3 patients with ET, who were kept awake during the implantation of DBS leads for intraoperative examinations for stimulation and adverse effects. Additionally, 4 patients who were enrolled in a trial exploring the effects of bilateral implantations of DBS leads for different nuclei (STN and GPi) in each hemisphere were counted.

**Table 1:** Demographic characteristics of included patients according to three lead anchoring devices

	Stimloc	TouchLoc	Traditional
Gender (F:M)	15:13	12:13	11:15
Age (Range)	59.2 ± 15.7 (18–79)	56.7 ± 14.9 (25–74)	54.5 ± 14.8 (17–78)
Nucleus	GPi (26), Vim (1) STN&GPi (1)	GPi (14), VC/VS (5) STN (2), STN&GPi (2) Hb (1), PSA (1)	GPi (22), Vim (1), Hb (1) PSA (1), STN&GPi (1)
Disease	PD (21), Dys (4) Tic (2), ET (1)	PD (12), Dys (3), Add (3), PS (3), ET (1), OCD (1), Anx (1), MDD (1)	PD (16), Dys (7) ET (2), MDD (1)
Manufacture	Medtronic (28)	SceneRay (25)	Medtronic (6), PINS (20)
Electrode	Unilateral (1) Bilateral (27)	Unilateral (1) Bilateral (24)	Unilateral (3) Bilateral (23)

* Numbers in parentheses are the specific subjects

*M* male, *F* female, *GPi* globus pallidus internal, *Vim* ventralis intermedius nucleus, *STN* subthalamic nucleus, *VC/VS* ventral capsule/ventral striatum, *Hb* habenular nucleus, *PSA* posterior subthalamic area, *PD* Parkinson’s disease, *Dys* primary/secondary dystonia, *Tic* tic disorder, *ET* essential tremor, *Add* drug addiction, *OCD* obsessive-compulsive disorder, *PS* Parkinsonian syndrome, *Anx* anxiety disorder, *MDD* major depressive disorder

### Lead measurement

**Fig. 3:**
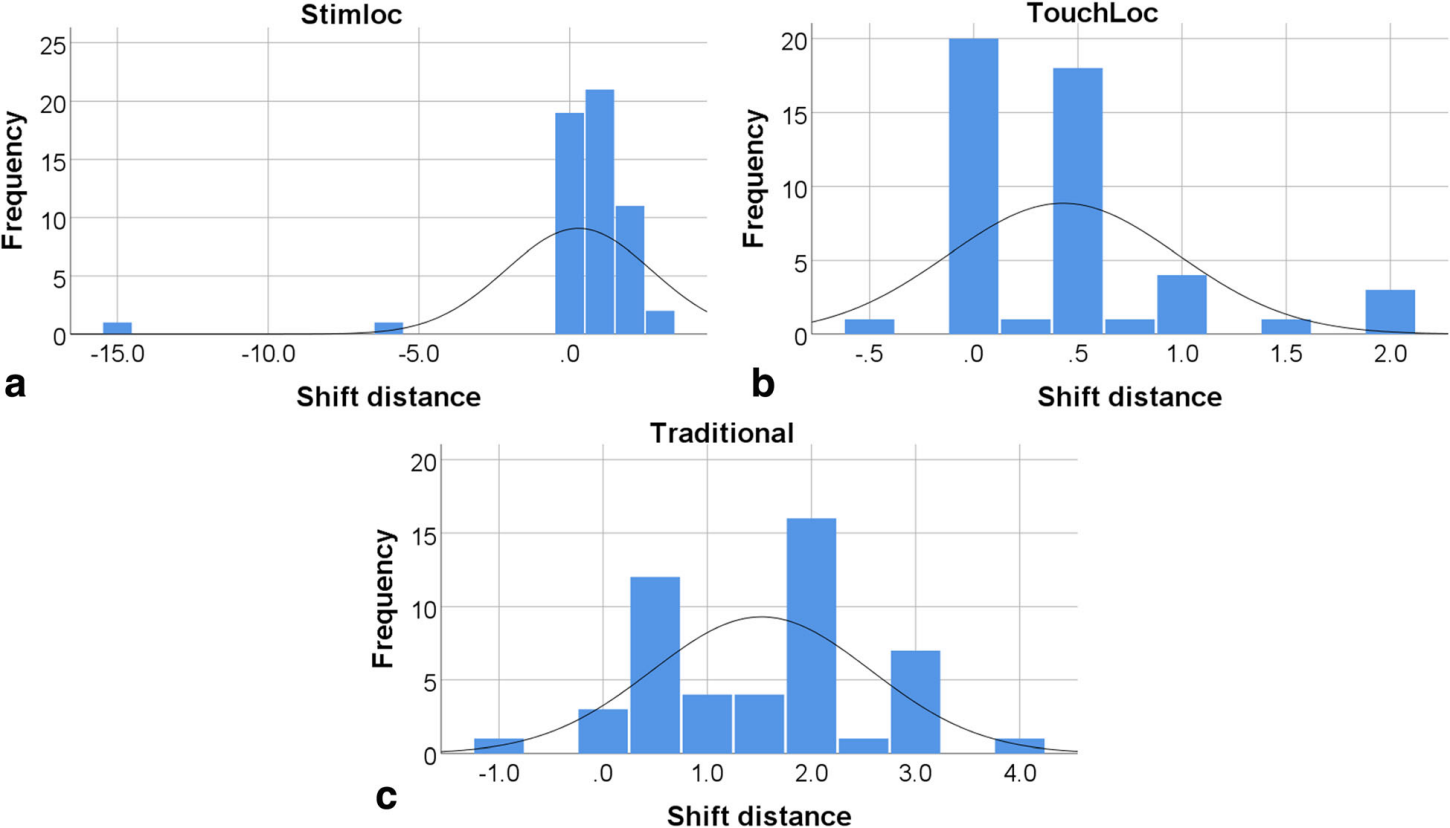
Frequency of lead shift distance according to lead anchoring device. **a**, Stimloc™^,^ (Medtronic, Minneapolis, MN, USA) device; **b**, TouchLoc (SceneRay, Suzhou, China) device; **c**, traditional anchoring device

In this series, most recorded distances of position shift were 0.5 mm (30.7% of all records) and 1.0 mm (15.7%). Although 0.0 mm (26.8%) was recorded in 34 patients with 41 lead implantations, the difference existed between the two images from C-arm fluoroscopy because of the allowed error range (Fig. 3). Comparison of the three lead anchoring devices revealed mean position shift distances of 0.29 ± 2.42 mm (Stimloc device [55 records]), 0.43 ± 0.55 mm (TouchLoc device [49 records]) and 1.52 ± 1.05 mm (traditional device [49 records]). The Kruskal-Wallis test revealed significant statistical differences among the three devices (test statistic = 34.593, *p* < 0.001). In this study, the Stimloc and TouchLoc devices demonstrated similar stable performance of lead anchoring compared to the traditional anchoring device. However, − 15.0 mm (once) and − 6.0 mm (once) were remarkably noticeable in shift distances of the Stimloc device. Furthermore, a few records (3.4%) yielded negative values of shift distance, indicating a practical trend toward a deeper location of lead than surgically planned.

**Fig. 4:**
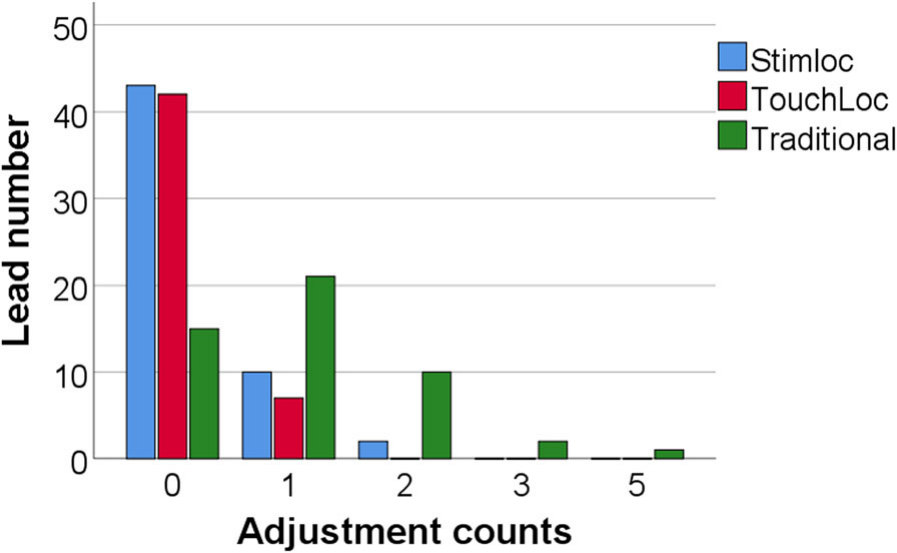
Frequency of lead adjustment according to lead anchoring device

As experience with DBS surgeries accumulated, the number of lead adjustments were usually limited to 0 (65.4%) or 1 (24.8%) by the time the surgical technique was fully developed (Fig. 4). The mean number of adjustments in this series were: 1.1 ± 1.0 (traditional), 0.3 ± 1.3 (TouchLoc), and 0.3 ± 0.5 (Stimloc). Meanwhile, a statistically significant difference was also revealed among the three devices using the Kruskal-Wallis test (test statistic = 42.905; *p < 0.001*), suggesting that lead adjustment counts for the traditional lead anchoring device were higher, regardless of manual factors.

The Kruskal-Wallis test was used to explore the main factors causing significant differences among the three devices. The difference in shift distances of electrode position was primarily caused by the less stable performance of the traditional anchoring device, while the Stimloc and TouchLoc devices demonstrated little difference (TouchLoc vs. Stimloc: test statistic = 9.280, *P* = 0.273; TouchLoc vs. Traditional: test statistic = − 48.082, *P* = 0.0001; Stimloc vs. traditional: test statistic = − 38.801, *P* < 0.0001). In contrast, the less stable performance of the traditional anchoring device led to the significant difference in lead adjustment times, whereas the Stimloc and TouchLoc devices demonstrated consistent performance in lead anchoring (TouchLoc vs. Stimloc: test statistic = 6.106, *P* = 0.403; TouchLoc vs. traditional: test statistic = − 45.112, *P* < 0.001; Stimloc vs. traditional: test statistic = − 39.006, *P* < 0.001).

## Discussion

Through the review of our patients who received DBS surgeries, with electrode positions confirmed by intraoperative C-arm fluoroscopy, we demonstrated the relatively stable performance in the newly designed lead anchoring device for lead fixation. Both patented devices have presented less lead shifts and adjustment counts than the traditional one. The details of the surgical protocols used in our center also provided feasible solutions for reducing the lead shifts and adjustment counts in such a sophisticated surgery.

Lead anchoring devices are currently available as three main products in the Chinese market. In the past two decades, traditional lead anchoring devices have played a vital role in lead securement by simply pressing the cap onto the dock, whereas the Stimloc (Medtronic) and TouchLoc (SceneRay) devices represent new generations of lead anchors. With their own proprietary design, these devices firmly fastened DBS leads and minimized manual error [11, 12]. In fact, in this particular series, use of the Stimloc and TouchLoc devices was associated with shorter lead shift distances and fewer adjustments than the traditional devices. So, it not only validated the lead fixation performance of the newer designs but supported the role of the anchoring device in DBS surgery as indispensable for better lead positioning. Moreover, our results exposed a basic flaw in the traditional lead anchoring devices. The essential function of lead securement was less stable when removing the lead guidewire and cannula for the first shift, pressing the lead on the fillister of the anchoring device for the second adjustment, and even putting the cap onto the dock for the third adjustment. If the three routine steps were not performed carefully, the strikingly shift distance (− 15.0 mm and − 6.0 mm) could be made in the Stimloc device because it was not firmly anchored.

Accumulated experience with DBS surgery resulted no or exceedingly low lead adjustment counts in one-half of the records. Nevertheless, lead fixation still needs careful operation as well as imaging confirmation, regardless of the manufactures of the lead anchoring device. Therefore, in addition to upgrading lead anchoring devices, surgical procedures have been gradually optimized for lead fixation in our center over the past decade. For example, when drilling a burr hole in the skull, special attention should be devoted to precisely centering the entry point hole for the DBS lead in the first step. Given the fabric nature of the Stimloc and TouchLoc devices, only the center of the dock would provide the maximal performance of lead fixation; therefore, a precisely centered burr hole is a worthy goal. In this condition, solid installation of the base ring on the burr hole and strictly following the surgical plan should be cautiously performed without much spare space left between the skull and the base ring. Once the implanted lead is in place, and after imaging to gauge the relative site of the electrode to the frame C-arm fluoroscopy, the true lock anchoring the lead is fitted onto the dock instead of manually holding the lead. Furthermore, once the lead is secured to the dock by the anchoring device, removing the lead guidewire and cannula should not cause any lead shifts, which suggests that large manual errors could also occur using the Stimloc and TouchLoc devices if the lock status is not cautiously verified.

Another subtle point that warrants attention is that the opening of the anchor may be at close to a right angle to the groove of the dock to reduce the potential shift when pressing the lead onto the dock. At that moment, a second C-arm fluoroscopy would be performed for verifying the effect of this press on lead shift, especially when a traditional lead anchoring device is applied. When the cap is pressed onto the dock to seal the traditional anchoring device, lead shift would also occur by extrusion of the cap with the lead and, as a result, a third round of imaging is needed for confirmation. Routinely, a second C-arm fluoroscopy could be performed when the cap of traditional lead anchoring device was pressed to simplify the workflow, and a second imaging would be sufficient for the other two types of anchoring devices. A point to be carefully noted is that the movement of the C-arm should be absolutely avoided during lead adjustments to avoid inappropriate image comparisons. Therefore, the results of this study were consistent with routine operations in our center in which most lead shifts (96.6%) suggested that the actual electrodes were deeper than the planned target, likely caused by the press of both the lead and the cap, whereas the shallower results were probably caused by unfavorable securement of the anchoring device.

In addition, selection of C-arm fluoroscopy as the primary intraoperative imaging method actually depends on workflow in that multiple examinations are required during DBS surgery, given that intraoperative CT and MRI are relatively time-consuming and, moreover, not popular in most Chinese functional neurosurgery centers. C-arm fluoroscopy can actively depict the depth deviation of electrode position during DBS implantation, which is easier to adjust within millimeters than in the other two dimensions. Several supplementary methods to reliably secure DBS leads have been reported in the recent literature, including applications of titanium microplates and bone cement [6, 13]. However, such intraoperative imaging methods remain indispensable for instant confirmation of electrode position in view of the potential lead shift by manual manipulation. As recorded, in addition to the first fluoroscopy following electrode implantation, the second imaging usually is performed with the guidewire and cannula removed, in which all three lead anchoring devices have any opportunities for lead shift, while the third fluoroscopy should be performed to verify the influence of the cap covering on the base ring in traditional lead anchoring devices. When lead adjustment is necessary, corresponding C-arm fluoroscopy to assess electrode position is required.

### Limitations

This study did have several limitations as follows regardless of the explicit results. First of all, the measurement of the lead shifts had inherent defects. The minimum unit of measurement was based on the length of the reference (the contacts of the DBS electrode and the gap between two contacts). Then the measurement could be not unified when different neurosurgeons measured those shifts manually through the images as Fig. 2 presented. Secondly, patients with various diseases received DBS implantations in different brain targets, which may have been a potential source of selection bias caused by the diverse shift trend of those targets in distinct brain depth, as well as of general brain conditions sorted according to age, disease, and other possible factors. Lastly, the significant differences among the three lead anchoring devices should not be interpreted as a reflection of the quality of these products. Surgical handling procedures and inevitable manual errors need special attention to the improvement of the accuracy in placement of DBS leads.

## Conclusions

Three lead anchoring devices have been compared for their performance in the accuracy of lead fixation, in which the newly designed lead fixation devices have presented its advantages to the traditional one. The lead shift caused by manual error still requires correction with intraoperative imaging to verify lead adjustment. Further studies aiming to improve surgical techniques that optimizing intraoperative lead shifts are needed.

## Data Availability

Available upon request to ldy11483@rjh.com.cn.

## Abbreviations

CT: Computed tomography
DBS: Deep brain stimulation
ET: Essential tremor
GPi: Globus pallidus internal
MRI: Magnetic resonance imaging
PD: Parkinson’s disease
STN: Subthalamic nucleus
